# Monounsaturated Fatty Acids and Risk of Cardiovascular Disease: Synopsis of the Evidence Available from Systematic Reviews and Meta-Analyses 

**DOI:** 10.3390/nu4121989

**Published:** 2012-12-11

**Authors:** Lukas Schwingshackl, Georg Hoffmann

**Affiliations:** Department of Nutritional Sciences, Faculty of Life Sciences, University of Vienna, Althanstrasse 14, 1090 Vienna, Austria; E-Mail: lukas.schwingshackl@univie.ac.at

**Keywords:** monounsaturated fatty acids, cardiovascular disease, coronary heart disease, meta-analysis, systematic review, dietary fat

## Abstract

No dietary recommendations for monounsaturated fatty acids (MUFA) are given by the National Institute of Medicine, the United States Department of Agriculture, European Food and Safety Authority and the American Diabetes Association. In contrast, the Academy of Nutrition and Dietetics, and the Canadian Dietetic Association both promote <25% MUFA of daily total energy consumption, while the American Heart Association sets a limit of 20% MUFA in their respective guidelines. The present review summarizes systematic reviews and meta-analyses of randomized controlled trials and cohort studies investigating the effects of MUFA on cardiovascular and diabetic risk factors, cardiovascular events and cardiovascular death. Electronic database Medline was searched for systematic reviews and meta-analyses using “monounsaturated fatty acids”, “monounsaturated fat”, and “dietary fat” as search terms with no restriction to calendar date or language. Reference lists and clinical guidelines were searched as well. Sixteen relevant papers were identified. Several studies indicated an increase of HDL-cholesterol and a corresponding decrease in triacylglycerols following a MUFA-rich diet. The effects on total and LDL-cholesterol appeared not consistent, but no detrimental effects on blood lipids were observed. Values for systolic and diastolic blood pressure were found to be reduced both during short- and long-term protocols using high amounts of MUFA as compared to low-MUFA diets. In type 2 diabetic subjects, MUFA exerted a hypoglycemic effect and reduced glycosylated hemoglobin in the long term. Data from meta-analyses exploring evidence from long-term prospective cohort studies provide ambiguous results with respect to the effects of MUFA on risk of coronary heart disease (CHD). One meta-analysis reported an increase in CHD events, however, most meta-analyses observed a lesser number of cases in participants subjected to a high-MUFA protocol. Although no detrimental side effects of MUFA-rich diets were reported in the literature, there still is no unanimous rationale for MUFA recommendations in a therapeutic regimen. Additional long-term intervention studies are required to characterized efficacy and effectiveness of recommending MUFA-rich diet among general and clinical populations.

## 1. Monounsaturated Fatty Acids

Monounsaturated fatty acids (MUFA) are chemically classified as fatty acids containing a single double bond (in contrast to polyunsaturated fatty acids (PUFA) containing two or more double bonds and saturated fatty acids (SFA) without double bonds). In the *cis*-configuration, the hydrogen atoms are on the same side as the double bond, whereas in *trans*-configuration the hydrogen atoms and the double bond are present on opposite sides. The *cis*-isomers are the predominant form of MUFA in food sources. The most common *cis*-configured MUFA in daily nutrition is oleic acid (18:1 *n*-9), followed by palmitoleic acid (16:1 *n*-7), and vaccenic acid (18:1 *n*-7). Moreover, oleic acid represents the topmost MUFA provided in the diet (~90% of all MUFAs) [1]. The major *trans*-configured MUFA is elaidic acid (*trans* 18:1 *n*-9). Some MUFA—such as mystrioleic (14:1 *n*-5), gondoic (20:1 *n*-9), erucic (22:1 *n*-9) and nervonic (24:1 *n*-9) acid—are synthesized in minor concentrations endogenously using other MUFAs as precursors (see Table 1 for a summary of different types of MUFA). Various sources for MUFA in food are given in Table 2 (for comparison, PUFA and SFA contents are given as well). The most frequently consumed MUFA rich dietary oils are canola and olive oil. Furthermore, over the last decade commercial production of high oleic acid modified dietary oils with improved stability for the use in food processing has been markedly increased in order to replace dietary oils rich in SFA and *trans* fatty acids [2]. It should be recognized that in some populations, MUFAs are provided in higher amounts in the form of erucic acid (C22:1 *n*-9), e.g., found in culinary oils derived from some *Brassica* spp. such as rapeseed and mustard seed [3]. It is therefore not surprising that due to their widespread occurrence in oils nuts, seeds, fruits and meat, the predominant source of MUFA is largely depending on individual dietary habits. Like other fatty acids, MUFA are almost completely absorbed by the intestine and are oxidized for energy production, converted into other fatty acids, or incorporated into tissue lipids.

**Table 1:** Selected monounsaturated fatty acids.

C-Atoms: Double Bonds	Scientific Name of Acid	Molecular Formula	Chemical Name
11:1	Undecylenic	C_10_H_19_COOH	*cis*-10-undecenoic acid
14:1	Myristoleic	C_13_H_25_COOH	*cis*-9-tetradecenoic acid
16:1	Palmitoleic	C_15_H_29_COOH	*cis*-9-hexadecenoic acid
16:1	Palmitelaidic	C_15_H_29_COOH	*trans*-9-hexadecenoic acid
16:1	/	C_15_H_29_COOH	*cis*-7-hexadecenoic
18:1	Petroselinic	C_17_H_33_COOH	*cis*-6-octadecenoic acid
18:1	Oleic	C_17_H_33_COOH	*cis*-9-octadecenoic acid
18:1	Elaidic	C_17_H_33_COOH	*trans*-9-octadecenoic acid
18:1	Vaccenic	C_17_H_33_COOH	*cis*-11-octadecenoic acid
20:1	Gondoleic	C_19_H_37_COOH	*cis*-9-eicosenoic acid
20:1	Gondolic	C_19_H_37_COOH	*cis*-11-eicosenoic acid
22:1	Cetoleic	C_21_H_41_COOH	*cis*-11-docosenoic acid
22:1	Erucic	C_21_H_41_COOH	*cis*-13-docosenoic acid
24:1	Nervonic	C_23_H45COOH	*cis*-15-tetracosaenoic acid

**Table 2:** Fatty acid content of different oils, nuts, fruits, seeds and animal products.

Oils	MUFA, %	PUFA, %	SFA, %
Olive oil	73	10.5	14
Coconut oil	6	2	86
Soybean oil	23	58	16
Peanut oil	46	32	17
Sesame oil	40	42	14
Sunflower oil (linoleic acid <60%)	45	40	10
High-oleic safflower oil	72	13	7.5
Sunflower oils (linoleic acid >70%)	14	75	6
Walnut oil	23	63	9
Almond oil	70	17	8
Hazelnut oil	78	10	7
Avocado oil	71	13	12
Canola oil	63	28	7
Mustard oil	59	21	12
High oleic sunflower	84	4	10
Hering oil	57	16	21
Fish oil, cold liver	47	23	23
Flaxseed oil, cold press	18	68	9
Corn and canola oil	58	29	8
High oleic sunflower	84	4	10
Hazelnut oil	78	10	7
Olive oil	73	10.5	14
High-oleic safflower oil	72	13	7.5
Avocado oil	71	13	12
Almond oil	70	17	8
Canola oil	63	28	7
Mustard oil	59	21	12
Corn and canola oil	58	29	8
Hering oil	57	16	21
Fish oil, cold liver	47	23	23
Peanut Oil	46	32	17
Sunflower Oil (linoleic acid <60%)	45	40	10
Sesame Oil	40	42	14
Soybean oil	23	58	16
Walnut oil	23	63	9
Flaxseed oil, cold press	18	68	9
Sunflower oils (linoleic acid >70%)	14	75	6
Coconut oil	6	2	86
**Nuts and Seeds**	**MUFA, %**	**PUFA, %**	**SFA, %**
Macademia	59	12	2
Hazelnut	46	8	4
Pecanut	41	22	6
Almonds	31	11	4
cashew nuts, dry roasted	27	7	9
Pistacchio nuts	24	14	5
Sunflower seed kernels, dried	19	23	4
Sesame, whole, roasted and toasted	18	21	7
Walnuts	15	35	3
Flaxseed	8	29	4
Safflower kernels, dried	5	28	4
**Products of Animal Origin**	**MUFA, %**	**PUFA, %**	**SFA, %**
Butter, salted	21	3	51
Cheese, cheddar	9	1	21
Pork, ham	8.3	2	6.5
Mackerl	5.4	3.3	3.2
Beef, steak	4.5	0.4	4.3
Egg	3.6	2	3
Salmon	2.1	2.5	0.9
Milk, 3.7% fat	1	0.1	2.2
Chicken	0.9	0.75	0.8

MUFA = monounsaturated fatty acid; PUFA = polyunsaturated fatty acid; SFA = saturated fatty acid [4].

## 2. Guidelines

### 2.1. General Nutrition Guidelines

Table 3 summarizes MUFA recommendations of national and international authorities and organizations.

**Table 3:** National and international MUFA recommendations for healthy adults and patients with diabetes.

Authority/Society	MUFA (% of TEC)	Target Group/Remarks	References
*American Heart Association*	<20	Healthy adults	[5]
*Academy of Nutrition and Dietetics/Canadian Dietetic Association*	<25	Healthy adults	[6]
*Dutch Dietary Guidelines*	8–38	Healthy adults Upper limit for obese: 25% of TEC	[7]
*European Food Safety Authority*	No specific recommendations	Healthy adults	[8]
*Italian Society of Human Nutrition*	No specific recommendations	Healthy adults	[9]
*Joint Committees of Germany, Austria, and Switzerland*	10	Healthy adults	[10]
*National Cholesterol Educational Program III*	<20	Healthy adults	[11]
*National Institute of Medicine*	No specific recommendations	Healthy adults	[12]
*Nordic Nutrition Dietary Guidelines*	10–15	Healthy adults	[13]
*Nutritional Recommendations for the French Population*	20	Healthy adults Including pregnant and lactating women	[14]
*UK COMA Committee*	12	Healthy adults	[15]
*US Department of Agriculture*	No specific recommendations	Healthy adults	[16]
*World Health Organization/Food Agriculture Organization*	15–20	Healthy adults Adjusted to total fat intake	[3]
*American Association of Clinical Endocrinologists*	No specific recommendations	Diabetic patients	[17]
*American Diabetes Association*	No specific recommendations	Diabetic patients Initial recommendation: 10%–20% of TEC	[18,19]
*British Diabetes Association*	10–15	Diabetic patients	[20]
*Canadian Diabetes Association*	No specific recommendations	Diabetic patients Replacement of SFA by MUFA	[21]
*European Association for the Study of Diabetes*	10–20	Diabetic patients Limitation of total fat to 35% of TEC	[22]
*International College of Nutrition of India*	7	Diabetic patients	[23]

MUFA = monounsaturated fatty acids; SFA = saturated fatty acids; TEC = total energy content.

In 1999, the International Society for the Study of Fatty Acids and Lipids agreed upon a recommendation table on daily intake of fatty acids as a foundation for further discussions. Adequate intake levels for adults were specified with respect to α-linolenic acid, eicosapentaenoic acid, docosahexaenoic acid, as well as upper limits for linoleic acid, *trans*-fatty acids, and saturated, given as % of total energy content (TEC), respectively. Given a total fat range from 15% to 40% of TEC, these recommendations included the suggestion to provide the majority of fatty acids in the form of MUFAs. However, no precise value (*i.e.*, % of TEC in the form of MUFA) was given by the panel [24]. According to the Joint FAO/WHO Expert Consultation Committee, MUFA intakes should be determined by calculating the difference: MUFA (% of TEC) = total fat (% of TEC) − SFA (% of TEC) − PUFA (% of TEC) − TFA (% of TEC). Accordingly, MUFA intakes (% of TEC) will range with respect to the total fat and fatty acid composition of the diet [3]. Based upon 13 peer-reviewed background papers dealing with fats and fatty acids in human nutrition, the Joint FAO/WHO Expert Consultation Committee concluded that replacement of carbohydrates by MUFA beneficially increases HDL-cholesterol, while the substitution of SFA with MUFA exerts favorable effects on LDL-cholesterol and the ratio of total cholesterol to HDL-cholesterol [3]. In their position on dietary fatty acids of 2007, the American and Canadian Dietetic Association suggested a high maximum quota of MUFA, *i.e.*, <25% of TEC [6]. Less than 20% of TEC should be consumed in the form of MUFA as recommended by the American Heart Association (AHA) in 2006, which is interesting with respect to the fact that the corresponding value was only <15% in the year 2000-statement of the AHA [5,25]. The National Cholesterol Education Program III suggested that <20% of TEC should be consumed in the form of MUFA [11]. In their Dietary Guidelines for Americans, edition 2010 [16], the United States Department of Agriculture (USDA) gives no specific recommendations for MUFA [16]. In addition, the National Institute of Medicine did not mention any specific recommendations for MUFA. In their statement, they concluded that “*n*-9 *cis* Monounsaturated fatty acids are synthesized by the body and have no known independent beneficial role in human health and are not required in the diet.” Therefore, neither an Adequate Intake nor a Recommended Daily Allowance was set. Since there is insufficient evidence for an Upper Level as well, the Dietary Reference Intakes did not consider MUFA at all [12]. In accordance with these proceedings and with a similar rationale, the European Food and Safety Authority (EFSA) skipped MUFA in their scientific opinions on dietary reference values for fat [8]. On a national level, the recommendations given in European countries are far from being conclusive. The Italian Society of Human Nutrition did not list any specific references for MUFA [9]. The Joint Committees of Germany, Austria, and Switzerland stated that MUFA consumption should be 10% of TEC, albeit with higher intakes being acceptable [10]. The Nordic Nutrition Recommendations agreed on 10%–15% of TEC in the form of MUFA [13]. The particulars of the Dutch Dietary Guidelines proposed a limit of 38% of TEC in the form of MUFA and PUFA for people with optimal body weight, whereas overweight and obese people should be more restrictive and limit their daily energy uptake in the form of MUFA/PUFA to 28% [7]. The UK COMA Committee advocated that MUFA (in the form of oleic acid) should provide an average of 12% of TEC [15]. The Nutritional Recommendations for the French Population promoted an intake of MUFA up to 20% of TEC for adults including pregnant and lactating women. It was emphasized that the neutrality of oleic acid represents a benefit and that its consumption was justified [14]. 

### 2.2. Specific Guidelines for the Prevention and Treatment of Diabetes

On closer examination, the MUFA recommendations of the American Diabetes Association (ADA) evolved in a “downhill” fashion. In 2002, a consumption of 10%–20% of TEC in the form of MUFA was proposed [18]. Two years later, a carbohydrate plus MUFA intake of 60%–70% of TEC was regarded as an evidence-based nutrition principle for the prevention and treatment of diabetes [26]. In 2008, the ADA’s position statement did not offer a specific value for MUFA as a preventive or therapeutic tool any longer [19]. Correspondingly, the American Association of Clinical Endocrinologists excluded MUFA in their medical guidelines for the management of diabetes [17]. However, the Canadian Diabetes Association suggested frequently replacing SFA with MUFA for a successful nutritional management of diabetes mellitus [21]. Likewise, the Joslin Diabetes Center recommended the consumption of MUFA, again without any specific reference values [27]. The Diabetes and Nutrition Study Group of the European Association for the Study of Diabetes stated that MUFA should provide 10%–20% of TEC with total fat to be limited to 35% of TEC [22]. The British Diabetes Association, probably referring to the 2004 nutrition principles of the ADA, recommended a daily amount of 60%–70% of TEC in the form of carbohydrates and MUFA, with MUFA values specified separately to aim at 10%–15% of TEC [20]. In Japan, no specific quota of MUFA is given in as a nutritional reference, while other Asian nations like India allow for up to 7% of TEC in the form of MUFA [23,28]. In South Africa, the corresponding authorities recommended <13% MUFA for diabetic subjects [29]. 

## 3. Risk Factors for Diabetes and Cardiovascular Disease

The National Cholesterol Education Program guidelines have outlined risk factors that increase CHD risk over a 10 year period. Elevated LDL-cholesterol (>100 mg/dL) remains the strongest primary factor in predicting CHD and therefore is a primary target of therapy [11]. However, as circulating triacyglycerols (TG) and HDL-cholesterol concentrations are critical risk factors in metabolic syndrome, the TC:HDL-cholesterol ratio has been expressed as a more valuable marker in determining CHD risk [30]. Summing-up, elevated levels of TC, LDL-cholesterol and TG as well low levels of HDL-cholesterol are evidence-based risk factors of CVD [31,32,33]. High levels of blood pressure are also associated with an increased mortality risk [34]. In addition, the Emerging Risk Factor Collaboration indicated FG levels >100 mg/dL as a predictor of mortality [35]. The Framingham Heart Study showed that impaired fasting glucose was associated with an aggravated risk of CHD in women [36]. A meta-analysis of cohort studies including 44,158 individuals without diabetes found a significant association between glycosylated hemoglobin (HbA1c) and cardiovascular events as well as death [37]. In another meta-analysis of observational studies, it was concluded that chronic hyperglycemia is associated with an increased risk of CVD in patients with type 2 diabetes mellitus (T2D) [38]. Among women, high-sensitive C-reactive protein (CRP) was the strongest predictor of CVD, accompanied by TC, LDL-cholesterol, TC:HDL-cholesterol, and Apo B 100 [39]. A recent meta-analysis indicated that Apo B is a more accurate marker of cardiovascular risk as compared to non-HDL-cholesterol (=TC-HDL-cholesterol), while the latter is still more accurate in comparison to LDL-cholesterol [40]. Strong associations between low serum HDL-cholesterol/high serum LDL-cholesterol and the onset of abdominal aortic aneurysms prove the continuous validity of both markers as predictive risk factors [41]. A collaborative analysis of individual data from 36 prospective studies involving more than 126,000 individuals, has demonstrated that circulating Lp(a) concentrations are correlated with an increased incidence of CHD and stroke independent from several conventional risk factors (including TC) [42]. 

## 4. Methods

### 4.1. Data Sources and Search Strategy

Electronic database MEDLINE (between 1966 and November 2012) was searched for systematic review and meta-analysis using following search terms “monounsaturated fatty acids”, “monounsaturated fat” and “dietary fat” with no restriction to calendar data and language. Reference lists and relevant clinical guidelines were also searched. 

### 4.2. Inclusion Criteria

Studies were included in this review if they met all of the following criteria: (1) systematic review/meta-analysis (quantitative analysis) including RCTs, crossover, metabolic, and observational studies; (2) intervention trials (isocaloric exchange): comparison of MUFA *vs.* carbohydrates, SFA, PUFA, and *trans*-fat; cohort studies: highest MUFA intake *vs.* lowest MUFA intake; (3) Study population: >18 years, healthy, patients with type 2 diabetes mellitus (T2D), obese, overweight; impaired glucose metabolism and cardiovascular disease (CVD); (4) outcome parameters: anthropometric outcomes, blood lipids, glycemic control parameters, blood pressure, inflammation markers and cardiovascular events/mortality.

### 4.3. Study Quality Assessment

Review quality was rated using a modified version of the Overview of Quality Assessment Questionnaire (OQAQ) including a bias tool [43] (Supplemental material, Table S1) as described recently [44]. Results of OQAQ assessments are summarized in Table 4. It should be noted that the analyses considered were in some cases based on overlapping sets of trials. 

**Table 4:** Qualitative aspects of the included systematic reviews and meta-analyses.

Reference	Aim	Methods (Inclusion/Exclusion criteria)	Heterogeneity	Period	Quality Assessment
Hegsted *et al.* 1993 [45]	Overall evaluation of the rather extensive literature on the effects of dietary fatty acid composition and cholesterol on serum lipid concentration	Design: metabolic studies (appear to have been done under rather careful control in which food was prepared and fed to the subjects); field trials (diet was modified by instructions or by a combination of instructions and provision of some foods)	not analyzed	until 1991	8
Mensink *et al*. 1992 [46]	Combining results to derive equations that relate changes in the dietary fatty acid intake to changes in serum HDL-C, LDL-C, TC and TG	Design: parallel design, crossover or Latin-square; “before and after” designs that lacked a control group were excluded. Diets enriched with very-long-chain ( *n*-3) PUFA were also excluded	not analyzed	1970–1991	10
Gardner *et al.* 1995 [47]	The purpose of this investigation was to address the controversy regarding a differential effect of MUFA *vs.* PUFA on serum lipids	Design: randomized trials comparing a high-mono and high-poly fat diet; similar in all respects (isoenergetic, total fat content, SFA) except for levels of monounsaturated and polyunsaturated fat intake; minimum 10 subjects on each diet arm	analyzed	1966–1994	12
Yu *et al.* 1995 [48]	Conducted to more comprehensively examine the effects of steraic acid, MUFAs, and other fatty acids on total and lipoprotein cholesterol concentrations in both men and women	Studies reported the quantity of individual SFA or steraic acid, sum of lauric, myristic and palmitic acids, and sum of MUFA and PUFA of the experimental diets.	not analyzed	1970–1993	8
		Exclusion. Liquid formula diets; diets that were specifically enriched with in *trans* isomers; diets enriched with very-long-chain PUFA; subject with familiar hypercholesterolemia			
Clarke *et al.* 1997 [49]	The aim of this meta-analysis of metabolic ward studies is to provide reliable quantitative estimates of the relevance of dietary intake of fatty acids and dietary cholesterol to blood concentrations of total cholesterol and cholesterol fraction	Design: dietary intervention studies conducted under controlled conditions that ensured compliance	not analyzed	/	9
Garg 1998 [50]	Examining the effects of high carbohydrate low fat diets *vs.* high MUFA diets on metabolic indexes in T2D subjects	Design: randomized, crossover trials using isoenergetic, weight maintaining diets	not analyzed	/	9
Mensink *et al.* 2003 [30]	Combining results to derive equations that relate changes in the dietary fatty acid intake to changes in serum HDL-C, LDL-C, TC and TG, Apo-B and Apo A-I, TC:HDL-C	Design: parallel design, crossover or Latin-square; “before and after” designs that lacked a control group were excluded. Diets enriched with very-long-chain ( *n*-3) PUFA were also excluded	not analyzed	1970–1998	13
Shah *et al.* 2007 [46]	Comparing high carbohydrate and high- *cis*-MUFA interventions trials conducted to increase understanding of the effect of carbohydrate and *cis*-MUFA on blood pressure	Design: randomized and non-randomized intervention studies comparing the effects of high-carbohydrate diets with those of high- *cis*-MUFA diets on blood pressure (crossover or parallel design), comparison of diets isoenergetic, body weight had to remain stable	analyzed	until 2006	12
Cao *et al.* 2009 [51]	Objective was to quantify the magnitude of the changes in lipids and lipoproteins in response to a MF blood cholesterol-lowering diet rich in unsaturated fat *vs.* LF in subjects with and without diabetes	Design: controlled feeding with a crossover or parallel design comparing MF *vs.* LF diets; designed to lower blood lipids; comparisons were isoenergetic; participants maintained constant weight during study; dietary protein and cholesterol were kept constant between diets	not analyzed	1987–2007	14
Jakobsen *et al.* 2009 [52]	Associations between energy intake from MUFA, PUFA, and carbohydrates and risk of CHD while assessing the potential effect-modifying role of sex and age	Design: cohort studies; published follow-up study with ≥150 incident coronary events; availability of usual dietary intake; a validation or repeatability study of the diet-assessment method used	analyzed	/	10
Kodama *et al.* 2009 [53]	To elucidate the effect of replacing dietary fat with carbohydrate on glucose and lipid parameters	Design: randomized controlled trials (crossover and parallel-group design); isoenergetic; only T2D	analyzed	1966–2007	16
		Exclusion: T1D, diets with change in in the content or quality of carbohydrates; heterogeneity analyzed			
Mente *et al.* 2009 [54]	Examining the association between nutrient intake, dietary components, and dietary patterns and CHD and its related clinical outcomes	Design: cohort studies; dietary pattern: higher intake level is compared with lowest intake level; *p*-values for trend, where available, were used to evaluate dose-response relationship. FFQ, food records, 24 h recalls; Bradford Hill criteria	analyzed	1950–2007	15
Mozaffarianand Clarke2009 [55]	Examining the effects on CHD risk of replacing partially hydrogenated formulations on other specific fats on the basis of the content of TFA, SFA, MUFA and PUFA	Design: randomized controlled trials (consumption of fatty acids on risk factors), cohort studies (association of habitual intake of fatty acids with incidence of CHD events); isocaloric replacement	not analyzed	until 2008	10
Skeaffand Miller2009 [56]	The purpose of this article was to summarize the evidence from cohort studies and randomized controlled trials of the relation between dietary fat and risk of CHD	Design: cohort studies; quintiles intake of PUFA, MUFA, SFA, TFA; The dietary assessment methods used in the cohort studies included single 24 h recall, diet records, diet histories and food frequency questionnaires; For MUFA only studies included in which exposure was determined by dietary assessment because blood fatty acids are not good biomarkers of MUFA intake	analyzed	/	10
Schwingshackl *et al.* 2011 [57]	Comparing high MUFA (>12% of TEC) *vs.* low MUFA (≤12% MUFA of TEC) on cardiovascular risk factors	Design: randomized controlled trials, ≥6 months, isocaloric and hypocaloric diets; subgroup analysis MUFA *vs.* LF, PUFA, LGI, HGI, Controls	analyzed	1966–2011	13
Schwingshackl *et al.* 2011 [58]	Comparing high MUFA (>12% of TEC) *vs*. low MUFA (≤12% MUFA of TEC) on glycemic control in subjects with abnormal glucose metabolism	Design: randomized controlled trials, ≥6 months, isocaloric and hypocaloric diets, subgroup analysis MUFA *vs.* LF, PUFA, LGI, HGI, Controls	analyzed	1966–2011	13

Apo A I: Apolipoprotein A-I; Apo B: Apo lipoprotein B; CHD: coronary heart disease; FFQ: food frequency questionnaire; HDL-C: high-density lipoprotein cholesterol; HGI: high glycemic index; LDL-C: low-density lipoprotein cholesterol; LF: low fat; LGI: low glycemic index; MF: moderate fat; MUFA: monounsaturated fat; PUFA: polyunsaturated fat; SFA: saturated fat; T2D: type 2 diabetes subjects; TC: total cholesterol; TEC: total energy content; TFA: *trans* fat; TG: triacylglycerols.

The present review included meta-analyses of intervention trials (randomized, non-randomized and crossover trials) and cohort studies. A common problem associated with cross-over trials is that of carry-over (a type of period-by-intervention interaction), but it seems only justifiable to exclude cross-over trials from a systematic review if the design is inappropriate within the clinical context [59]. Duration of studies varied remarkably between the different meta-analyses as well as between the different within each meta-analysis. This represents a major problem especially when comparing intervention trials. Sensitivity analyses comparing short- *vs.* long-term studies might be used as an alternative approach to resolve this issue. Another issue associated with meta-analyses is heterogeneity of various aspects and characteristics of the study protocols, especially in nutritional intervention trials. Therefore, it is not surprising that the literature chosen for the present review varies regarding type(s) of diets used (MUFA *vs.* carbohydrates/PUFA/SFA/*trans* fatty acids), definitions of MUFA diets, and study population (healthy, overweight, or obese subjects, patients with T2D, abnormal glucose metabolism, or CVD). In addition, in most of the included meta-analyses differential compliance (drop outs) was not investigated. Another potential source of bias is measurement issues (especially of self-reported data, e.g., 24 h recalls, food records). Only few systematic reviews screened for the presence of publication bias by assessing the symmetry of the funnel plots in which mean differences were plotted against their corresponding standard errors. 

## 5. Evidence from Meta-Analyses

### 5.1. Healthy Subjects

See Table 5 summarizes the study characteristics of the meta-analyses included in this review. For a better understanding of the categorization of meta-analyses and other scientific studies, the Levels of evidence by the Scottish Intercollegiate Guidelines Network are given in Table 6 [60].

**Table 5:** Study characteristics of meta-analyses.

Reference	No. Studies	Statistical Method	Min. Duration	Participants	Effects of MUFA
Hegsted *et al*. 1993 [45]	*n* = 77	Multiple regression	n.d.	n.d.	↔ TC, LDL-C, HDL-C
Mensink *et al*. 1992 [61]	*n* = 28	meta-regression	14 days	682	↓ TG, HDL-C:LDL-C
					↑ HDL-C
					↔ TC, LDL
Gardner *et al*. 1995 ** [47]	*n* = 14	Standardized effect size	3 weeks	439	↑ TG *
					↔ LDL-C, HDL-C
Yu *et al.* 1995 [48]	*n* = 18	Meta-regression analysis	n.d.	804	↓ TC, LDL-C
					↑ HDL-C
Clarke *et al.* 1997 [49]	*n* = 91	Multilevel regression analysis	2 weeks	5910	↑ HDL-C
					↔ TC, LDL-C
Garg 1998 [50]	*n* = 9	meta-analysis	2 weeks	133	↓ TG, TC, VLDL-C, FG
					↑ HDL-C, Apo A-1
					↔LDL-C, Apo B, FI, HbA1c
Mensink *et al*. 2003 [30]	*n* = 60	meta-regression	13 days	1672	↓ TG, LDL-C, Apo B, TC:HDL-C
					↑ HDL-C, Apo A-1
					↔ TC
Shah *et al.* 2007 [46]	*n* = 10	Random effect modell	3 weeks	400	↓ SBP, DBP *
Cao *et al.* 2009 [51]	*n* = 30	Random effect modell	2 weeks	1213	↓ TG
					↑ HDL-C, Apo A 1
					↔ LDL-C
Jakobsen *et al*. 2009 [52]	*n* = 11	Random effect meta-analysis	4 years	344,696	↑ risk of CHD events
					↔ risk of CHD death
Kodama *et al*. 2009 [53]	*n* = 11	Fixed effect modell	10 days	329	↓TG
					↔ FG, FI, TC, HDL-C, LDL-C
Mente *et al.* 2009 [54]	*n* = 146	Random effect meta-analysis	n.d.	101,521	↓ CHD events
Mozaffarian and Clarke 2009 [55]	*n* = 13	Multilevel regression analysis	2 weeks	554	↓ TC, TG, LDL-C, Apo B, TC:HDL-C
					↑ HDL-C, Apo A-1
Skeaff *et al.* 2009 [56]	*n* = 28	Random effect meta-analysis	4 years	280,000	↔ risk of CHD death/events
Schwingshackl *et al.* 2011 [57]	*n* = 12	Random effect meta-analysis	6 months	1990	↓ FM, SBP, DBP
					↔W, WC, TC, LDL-C, HDL-C, TG, CRP
Schwingshackl *et al.* 2011 [58]	*n* = 9	Random effect meta-analysis	6 months	1547	↓ HbA1c, FG
					↔ FI, HOMA-IR

↑ significant increase; ↓ significant decrease; ↔ no significant effects; * *p* = 0.05; ** MUFA *vs.* PUFA; MUFA/PUFA for SFA decrease LDL-Cholesterol; n.d.: no data.

**Table 6:** Levels of evidence by the Scottish Intercollegiate Guidelines Network.

1++ High quality meta-analyses, systematic reviews of RCTs, or RCTs with a very low risk of bias	
1+ Well conducted meta-analyses, systematic reviews, or RCTs with a low risk of bias	
1− Meta-analyses, systematic reviews, or RCTs with a high risk of bias	
2++ High quality systematic reviews of case control or cohort studies	
High quality case control or cohort studies with a very low risk of confounding or bias and a high probability that the relationship is causal	
2+ Well conducted case control or cohort studies with a low risk of confounding or bias and a moderate probability that the relationship is causal	
2− Case control or cohort studies with a high risk of confounding or bias and a significant risk that the relationship is not causal	
3 Non-analytic studies, e.g., case reports, case series	
4 Expert opinion	
A	At least one meta-analysis, systematic review, or RCT rated as 1++, and directly applicable to the target population; or
	A body of evidence consisting principally of studies rated as 1+, directly applicable to the target population, and demonstrating overall consistency of results
B	A body of evidence including studies rated as 2++, directly applicable to the target population, and demonstrating overall consistency of results; or
	Extrapolated evidence from studies rated as 1++ or 1+
C	A body of evidence including studies rated as 2+, directly applicable to the target population and demonstrating overall consistency of results; or
	Extrapolated evidence from studies rated as 2++
D	Evidence level 3 or 4; or Extrapolated evidence from studies rated as 2+

In their meta-analysis, Clarke *et al.* (1997) [49] investigated the effects of MUFA as well as SFA and PUFA on cardiovascular risk factors in non-diabetic subjects. In addition, liquid formula diets were included, although they were analyzed separately. Dietary protocols were mostly *iso*-energetic but differed with respect to study design: they included randomized crossover, randomized or matched parallel, non-randomized Latin square and non-randomized sequential attempts. The authors concluded that substitution of carbohydrates by MUFA (5% of TEC) had no significant effect on TC and LDL-cholesterol, but managed to increase HDL-cholesterol. With respect to PUFA-rich diets, TC and LDL-cholesterol were both decreased and HDL-cholesterol was augmented in solid food experiments [49]. Clarke and Mozaffarian (2009) [55] observed that replacing hydrogenated fats with MUFA (1% of TEC) resulted in advantageous changes of several CVD risk factors like TC, LDL-cholesterol, HDL-cholesterol, TG, apoproteins A-1, B as well as B/A1, and lipoprotein (a) in 12 crossover and 1 parallel designed trials. Yu and co-workers (1995) [48] explored the results of 18 studies (again including crossover and parallel designed set-ups) enrolling a total of 804 healthy and normocholesterolemic participants. Following meta-regression, they observed that MUFA increased HDL-cholesterol and decreased TC and LDL-cholesterol. The corresponding effects of PUFA were more pronounced with respect to TC and LDL-cholesterol, but not to HDL-cholesterol [48]. In 1992, a meta-analysis of short-term RCTs investigated the effects of dietary fatty acids as an *iso*-caloric substituent for carbohydrates on CVD risk factors. HDL-cholesterol levels were significantly augmented following the MUFA-rich diet, while levels of TG and the ratio of TC to HDL-cholesterol were significantly reduced, respectively [61]. In 2003, the authors published an updated meta-analysis including 1672 instead of 682 participants and were able to confirm their previous results. In addition, they observed a significant improvement in LDL-cholesterol, Apo A-1, and Apo B following high-MUFA regimens [30]. In a recent meta-analysis investigating the long-term (≥6 months) effects of high- (>12% MUFA) *vs.* low- (≤12% MUFA) MUFA diets on cardiovascular risk factors, we could show that high-MUFA diets significantly reduced systolic and diastolic blood pressure in overweight/obese subjects [57] thus confirming data previously reported by Shah *et al*. in 2007 [46]. When MUFA-rich diets were compared with PUFA-rich onsets, no effects on HDL-cholesterol and LDL-cholesterol, but a borderline increase (*p* = 0.05) in TG could be observed [47]. Hegsted *et al.* [45] analyzed metabolic studies and field trials and could not observe any impact of MUFA on TC, LDL-cholesterol, and HDL-cholesterol in their meta-regression.

### 5.2. Patients with Abnormal Glucose Metabolism/Diabetes Mellitus

In a recent meta-analysis of short-term RCTs (crossover and parallel study designs) with a duration between 10 days and 6 weeks enrolling 306 subjects with type 2 diabetes mellitus, a significant decrease in TG values following a MUFA-rich dietary regimen could be observed when compared with a low-fat/high carbohydrate diet [53]. This is in congruence with data presented by Garg (1998) [50] reporting reduced fasting TG in patients with type 2 diabetes mellitus subjected to a weight maintenance diet following replacement of carbohydrates by MUFA [50]. Moreover, improvements in FG and pre-prandial plasma glucose were shown, while no significant changes in fasting plasma insulin concentrations, fructosamine and HbA1c were observed. The high-MUFA protocols were accompanied with significantly lower values for TC and VLDL-cholesterol as well as increases in HDL-cholesterol, but were not correlated to changes in LDL-cholesterol. Comparison of high- (>12% MUFA) *vs.* low- (≤12% MUFA) MUFA diets on glycemic control in subjects with abnormal glucose metabolism revealed improvements in HbA1c and fasting glucose in diabetic subjects, but no differences in blood lipids were found [58,62]. 

With respect to short-term studies (2–12 weeks duration), comparison of low *vs.* moderate dietary fat content was performed in a meta-analysis by Cao *et al.* (2009) [51]. Participants with and without diabetes and a body mass index ranging from 21.1 to 30.2 kg/m^2^ were enrolled. The mean MUFA content in a correspondingly modified diet was 23.6% of TEC and 11.4% in the low-fat versions. In the healthy collective, HDL-cholesterol was significantly increased and TG levels were significantly decreased in the moderate fat groups as compared to low-fat diets. TC and LDL-cholesterol were reduced in a similar fashion following both dietary protocols (moderate and low fat). Patients with diabetes adopting the diet with a higher MUFA content established a significant increase in HDL-cholesterol as well, accompanied by a significant reduction in TG and a non-significant reduction in TC as compared to the low fat diets. TG response was even more pronounced in participants with diabetes as compared to healthy subjects [51]. 

### 5.3. Patients with CVD

In a prospective trial investigating the effects of a Mediterranean diet, the Lyon Diet Heart Study reported a benefit of increased MUFA intake in survivors first time myocardial infarction [63].Three recent meta-analyses of cohort studies investigated the effects of dietary fats on CHD events and cardiovascular death. Skeaff and Miller [56] did not observe any effects of MUFA-rich diets on relative risks of CHD events and death. Moreover, no differences between of high- and low-fat intake were registered [56]. Jakobsen [52] performed a meta-analysis of cohort studies including 344,696 subjects. They postulated a positive correlation between MUFA-rich diets and risk of coronary events, but not between MUFA-rich diets and risk of coronary deaths. The authors explain that in the western diet, the MUFA supply is predominantly of animal origin resulting in a confounder that should be taken into consideration when comparing dietary fats. The usual source of MUFA/oleic acid is of vegetable origin. These results are in strong discrepancy with another recent meta-analysis of cohort studies, were Mente *et al.* [54] reported a correlation between MUFA uptake and a significant decrease in the relative risk for CHD. None of these three meta-analyses reported information regarding stroke or arrhythmic diseases, but included data for “hard” CHD endpoints like angina pectoris, sudden death, fatal and non-fatal myocardial infarction. In June 2012, the Cochrane Collaboration updated their systematic review and meta-analysis on the effects of low *vs.* modified fat diets on cardiovascular disease. The findings are suggestive of a small but potentially important reduction in cardiovascular risk on modification of dietary fat (but not reduction of total fat) in longer trials. However, no association between total fat content and risk of cardiovascular death and events were reported [64]. 

## 6. Conclusions

In comparison, a considerably larger number of meta-analyses explored the effects of PUFAs on maintenance or reduction of body weight as well as biomarkers of impaired glucose metabolism or CVD/CHD than there are systematic reviews and meta-analyses dealing with the corresponding impact of MUFAs. Consequently, the international recommendations for PUFA are more consistent than those for MUFA, averaging a value of 10% of TEC for healthy persons for the most part. If MUFA recommendations are given at all, they vary between 12% and 25% of TEC, equaling a remarkable range of ~30–70 g/day for a 2.500 kcal-diet. Prestigious authorities and organizations such as the National Institute of Medicine, the EFSA, the USDA and the ADA do not provide specific recommendation for MUFAs either for healthy people or for patients in need of diabetic or cardiovascular management. 

In the present review, only meta-analyses were included, which indicates a high level of evidence, *i.e.*, from 2+ to 1+++ according to the Scottish Intercollegiate Guidelines Network indicate levels of evidence (Table 6). Apart from the fact that several meta-analyses and meta-regressions observed benefits of MUFA on cardiovascular risk factors, it should be noted that most meta-analyses did not report significant negative effects of a MUFA-rich diet on CVD risk factors. With respect to the favorable influences of MUFA found in studies recruiting healthy volunteers or patients with diabetes and CHD respectively, some reservations still remain. Due to various inhomogeneities, the results of different studies are far from being conclusive. Thus, MUFA were compared to carbohydrate-rich diets, low fat diets or regimens focusing on PUFA or SFA. Moreover, the term MUFA-rich diet lacks a concrete definition leading to inconsistent amounts of MUFA used in the corresponding protocols. Some of the discrepancies in the findings of different studies can be explained by their uneven and maybe incompatible durations. Long-term biomarkers of glucose metabolism such as HbA1c will be most likely not or just slightly improved following short-term interventions of 2–6 weeks Nevertheless, in view of the importance of dietary interventions for the prevention and therapy of cardiovascular disease, monounsaturated fatty acid may represent a valuable tool in the modification of dietary regimens. There is strong evidence that by replacing SFA and carbohydrates with MUFA, various cardiovascular risk factors will be significantly improved. The results of the different meta-analyses addressed in this review point to a beneficial effect of MUFA-rich diets on systolic and diastolic blood pressure as well as parameters of glycemic control. On the other hand, the impact of MUFA on blood lipids is still discussed controversially. While TG levels were decreased and HDL-cholesterol levels were increased following short-term interventions with higher amounts of MUFA, these findings could not be confirmed in long-term study protocols. Thus, there is no unanimous rationale for MUFAs in a therapeutic regimen. However, since no detrimental effects of MUFA-rich diets were reported in the literature to date, there is no evidence speaking against the consideration of MUFAs in dietary guidelines. Further studies dealing with long-term effects of MUFA on biomarkers of obesity, diabetes, and cardiovascular diseases as well as clinical endpoints are needed to clarify the potential benefits of MUFA-rich diets in primary and secondary prevention.

## Supplementary Files

Supplementary File 1
