# Supplementary material for: Monounsaturated Fatty Acids and Risk of Cardiovascular Disease: Synopsis of the Evidence Available from Systematic Reviews and Meta-Analyses
Source: Nutrients. 2012 Dec 11;4(12):1989–2007. doi: 10.3390/nu4121989 (PMC3546618; doi:10.3390/nu4121989)
Supplement: Supplementary File 1 [file nutrients-04-01989-s001.pdf]

Supplementary Information

Table S1. OQAQ: Quality assessment tool for systematic reviews and meta-analyses. A modified version of the OQAQ was used to assess the quality of reviews. This consists of the following nine questions each answerable as "yes", "no" or "partially/can't tell", carrying scores of 2, 0 and 1, respectively.

**1. Were the search methods used to find evidence on the primary question(s) stated?**
(a) *Yes*, description of databases searched, search strategy, and years reviewed. *Two points*.<br>(b) *Partially*, descriptions of methods not complete. *One point*.<br>(c) *No*, no description of search methods. *No points*.
**2. Was the search for evidence reasonably comprehensive?**
(a) *Yes*, at least one computerised database searched and also a search of unpublished or non-indexed literature. *Two points*.<br>(b) *Can't tell*, search strategy partially comprehensive, at least one of the strategies performed. *One point.*<br>(c) *No*, search not comprehensive or not described well. *No points*.
**3. Were the criteria used for deciding which studies to include in the review reported?**
(a) *Yes*, inclusion and exclusion criteria clearly defined. *Two points*.<br>(b) *Partially*, reference to inclusion and exclusion criteria can be found but are not defined clearly enough. *One point*.<br>(c) *No*, no criteria defined. *No points*.
**4. Was bias in the selection of articles avoided?**
(a) *Yes*, issues influencing selection bias were covered. Both of the following bias-avoiding strategies were used: (1) two or more assessors independently judged study relevance, (2) assessors selected studies using predetermined criteria. *Two points*.<br>(b) *Can't tell*, only one of the strategies used. *One point*.<br>(c) *No*, selection bias was not avoided or was not discussed. *NO points*.
**5. Were the criteria used for assessing the methodological quality of studies reviewed reported?**
(a) *Yes*, criteria defined and used addressed the major factors influencing bias. *Two points*.<br>(b) *Partially*, some discussion or reference to criteria. *One point*.<br>(c) *No*, validity or methodological quality criteria not used or not described. *No points*.
**6. Were study quality assessment criteria used to inform the review analysis?**
(a) *Yes*, criteria were used to inform the analysis, either by exclusion from the analysis if low quality or through sensitivity analysis. *Two points*.<br>(b) *Partially*, some discussion but not clearly described application of criteria. *One point*.<br>(c) *No*, criteria not used or not described. *No points*.
**7. Were the methods used to combine the findings of the relevant studies (to reach a conclusion) reported?**
(a) *Yes*, qualitative and quantitative methods are acceptable. *Two points*.<br>(b) *Partially*, partial description of methods to combine and tabulate; not sufficient to duplicate. *One point*.<br>(c) *No*, methods not stated or described. *No points*.

Table S1. *Cont.*

**8. Were findings of the relevant studies combined appropriately relative to the primary question of the overview?**
(a) *Yes*, combining of studies appears acceptable. *Two points*.
(b) *Can't tell*, should be marked if in doubt. *One point*.
(c) *No*, no attempt was made to combine findings, and no statement was made regarding the inappropriateness of combining findings. *0 points*.
**9. Were the conclusions made by the author(s) supported by the data and/or analysis reported in the overview?**
(a) *Yes*, data were reported that support the main conclusions regarding the primary question(s) that the overview addresses. *Two points*.
(b) *Partially*. *One point*.
(c) *No*, conclusions not supported or unclear. *0 points*.
